# Orthopædic Surgery

**Published:** 1901-03

**Authors:** 


**Orthopædic Surgery.** By CHARLES BELL KEETLEY. Pp. xvii., 539. London: Smith, Elder, & Co. 1900.

Mr. Keetley's work on orthopædic surgery has appeared at a time when many such books have been published. He tells us, however, that he planned the work twenty years ago, and that though he recognises that now there is no great want for such a book, he still thinks that his observations in orthopædic surgery, extending over a period of more than twenty years, should now be published. And clearly the book is the outcome of much original thought and extended observation. Mr. Keetley is a surgeon who devotes a great amount of energy to the elucidation of any branch of surgery in which he may be interested, and he has undoubtedly done so with regard to orthopædics. The book is profusely illustrated. Many of the pictures are good, but several of the photographs are hardly worthy of the book. The order of the chapters is not always well arranged. Why should the chapter on rickets be placed between that on genu valgum and the one on bow legs? It should have preceded both.

The first deformity considered is that of genu valgum. The writer reproduces many of the current views as to its pathology, but gives no evidence that he has himself investigated it, either by dissection of deformed limbs, the examination of skiagrams, or the study of a large number of museum specimens. He does give a figure of one museum specimen, but from the extreme rotation of the limb inwards and the anterior curve of the femur, it is clear that it is not an ordinary case of genu valgum. We notice that the author states his belief that the tibia is more often at fault than some surgeons admit, and that Macewen exaggerates the degree and importance of the elongation of the internal condyle, but he brings no evidence in support of these statements. We observe that Mr. Keetley is opposed to Phelps's operation for equino-varus. Phelps has so modified his original operation that it is best to define exactly what we mean by Phelps's operation. It is the free division of the skin Mr. Keetley so strongly objects to. Why we fail to see. Our experience is, that if the case is at all a severe one, only when we get a wide gap in the divided skin can we thoroughly correct the deformity; and surely it is better to see what we are about when we divide the contracted muscles, tendons and ligaments in the sole, than to cut blindly with a tenotome. After objecting to Phelps's operation, the author writes with regard to cuneiform tarsectomy: "Very good results may be attained by the wedge operation, provided only that the soft structures in the concavity at the inner side of the foot, including the ligaments and tendons, and even the skin if necessary, be divided as freely as each case may demand." This is practically a description of Phelps's operation as that surgeon now performs it, for in severe cases he combines a division of the contracted soft parts with a division of the neck of the astragalus or the removal of a wedge of bone.

Coxa vara, of course, finds a place in this work, as it would in any recent book on orthopædic surgery. The author claims that he was one of the earliest surgeons to describe the disease, but we think that he hardly substantiates this claim. The case he published (with a drawing and brief description) was not apparently an ordinary case of coxa vara, as there was a marked subtrochanteric curve.

We are glad to see that in his treatment of wry neck, due to the contraction of the sterno-mastoid, Mr. Keetley says he refuses to follow the advice of those who recommend subcutaneous division of bands of fascia beneath the muscle, but would rather deal with them by the open incision. It is the frequent need for the division of these bands which constitutes one strong argument in favour of the method by open incision.

The author has, we think wisely, not included diseases of joints in his book, a practice usual with writers on orthopædic surgery in America; and he tells us that he has even abandoned his original intention of including ankylosis of joints among the other subjects treated of, for he considers that this necessitates a consideration of those diseases of the joints which may lead to ankylosis.

